# Antibiotic Over-Prescription by Dentists in the Treatment of Apical Periodontitis: A Systematic Review and Meta-Analysis

**DOI:** 10.3390/antibiotics13040289

**Published:** 2024-03-22

**Authors:** Juan A. Méndez-Millán, María León-López, Jenifer Martín-González, Juan J. Saúco-Márquez, Daniel Cabanillas-Balsera, Juan J. Segura-Egea

**Affiliations:** Department of Stomatology (Endodontic Section), School of Dentistry, University of Sevilla, C/Avicena s/n, 41009 Sevilla, Spain; jamendezmillan@hotmail.com (J.A.M.-M.); maria.leon.lopez.98@gmail.com (M.L.-L.); jmartin30@us.es (J.M.-G.); jjsauco@us.es (J.J.S.-M.)

**Keywords:** antibiotics, apical abscess, apical disease, apical periodontitis, endodontic infection, meta-analysis, periapical disease, prescription, systematic review

## Abstract

After pulp infection and necrosis, the passage of microbial antigens into the periapical space causes apical periodontitis (AP). Most of the clinical forms of AP can be managed without prescribing antibiotics, only with root canal treatment and abscess drainage or, where appropriate, tooth extraction. However, the scientific literature provides evidence of inappropriate antibiotic prescriptions by dentists in the management of apical disease. Objectives: The aim of this systematic review and meta-analysis was to analyze the global pattern of antibiotic prescription in the treatment of apical disease. Methods: PRISMA Guidelines were followed to carry out this systematic review. The research question was as follows: What is the pattern of antibiotic prescription by dentists in the treatment of the different clinical forms of apical periodontitis? A systematic search was conducted on MEDLINE/PubMed, Wiley Online Database, Web of Science and Scopus. All studies reporting data about the pattern of antibiotic prescription by dentists in the treatment of apical disease were included. The meta-analyses were calculated using the Open Meta Analyst version 10.10 software. Random-effects meta-analyses were performed. The risk of bias was assessed using the Newcastle–Ottawa Scale. The certainty of evidence was assessed using GRADE. Results: The search strategy identified 96 articles and thirty-nine cross-sectional studies fulfilled the inclusion criteria. The overall percentage of antibiotic prescriptions by dentists in cases of symptomatic AP was 25.8%, and 31.5% in cases of asymptomatic AP with sinus tract present. The percentage of dentists prescribing antibiotics in cases of acute apical abscess with no/mild symptoms was 47.7%, whereas, in cases of acute apical abscess with moderate/severe symptoms, 88.8% of dentists would prescribe antibiotics. Endodontists prescribe antibiotics at a lower rate than general practitioners. The total risk of bias was considered moderate, and the final rating for the certainty of the evidence was low. Conclusions: Dentists worldwide are over-prescribing antibiotics in the management of apical disease. It is necessary to improve antibiotic prescribing habits in the treatment of endodontic infections, as well as educational initiatives to encourage the rational and appropriate prescription of antibiotics in periapical diseases.

## 1. Introduction

The infection and necrosis of the pulp, with the subsequent passage of antigens into the periapical space, cause endodontic periapical disease [1]. A rapid onset, spontaneous pain, the extreme sensitivity of the tooth to pressure, and edema of the affected tissues characterize the periapical acute inflammatory reaction, named symptomatic apical periodontitis [2]. Without treatment, symptomatic apical periodontitis evolves into an acute apical abscess, with an accumulation of purulent discharge in the periapical space, swelling, extrusion of the tooth from the alveolus, abnormal mobility, and finally dissemination, causing osteomyelitis and cellulitis [3], potentially leading to the death of the patient [4]. However, a fatal outcome is fortunately uncommon and is usually associated with an immunocompromised status. Currently, the development and application of new diagnostic and therapeutic technologies in endodontics, as well as the use of antibiotics when indicated, have reduced mortality due to systemic dissemination of apical abscesses to almost zero.

Treatment of symptomatic apical periodontitis and acute apical abscess requires achieving adequate drainage by opening the pulp chamber. When the purulent discharge has destroyed the periosteum and the abscess is already in the submucosal phase, causing swelling of the intraoral soft tissues, it must be incised with a scalpel blade to establish drainage [5]. If the patient is immunocompetent, this intervention resolves the clinical picture. However, in immunocompromised patients, or when there are systemic symptoms or the purulent discharge progresses and expands, the prescription of systemic antibiotics is indicated [6]. On the contrary, asymptomatic apical periodontitis and chronic apical abscesses do not require antibiotic treatment [6,7].

Since the discovery of antibiotics and their introduction into clinical practice in the 1940s, antibiotics have been used in the treatment of endodontic infections, both systemically and topically [8,9]. Since then, bacteria, both pathogenic and commensal saprophytes, have been exposed to antibiotics. At first, they were sensitive to them, but over time, they have developed resistance [10]. Considering that dentists prescribe approximately 10% of all common antibiotics, the impact of dentists on antimicrobial resistance can be considerable [11]. A survey carried out in the UK in 2004 revealed that 40% of general dental practitioners prescribed three antibiotics each week, and 15% prescribed them on a daily basis [10]. Literature provides evidence of inadequate prescribing practices by dentists for a number of factors, ranging from inadequate knowledge to social factors [12,13,14]. Moreover, several surveys carried out with dentists from different countries have shown that antibiotics are being prescribed inappropriately in the treatment of periapical pathology [13,15,16,17,18,19,20]. To improve the prescription of antibiotics in the treatment of endodontic infections, the European Society of Endodontology (ESE) published an official position based on scientific evidence established by a committee of experts [6,9]. In addition, awareness campaigns led by dental and medical associations have been carried out throughout the world.

This study aims to carry out a systematic review with meta-analysis to investigate the global pattern of antibiotic prescription by dentists in the treatment of apical disease.

## 2. Results

**Figure 1:**
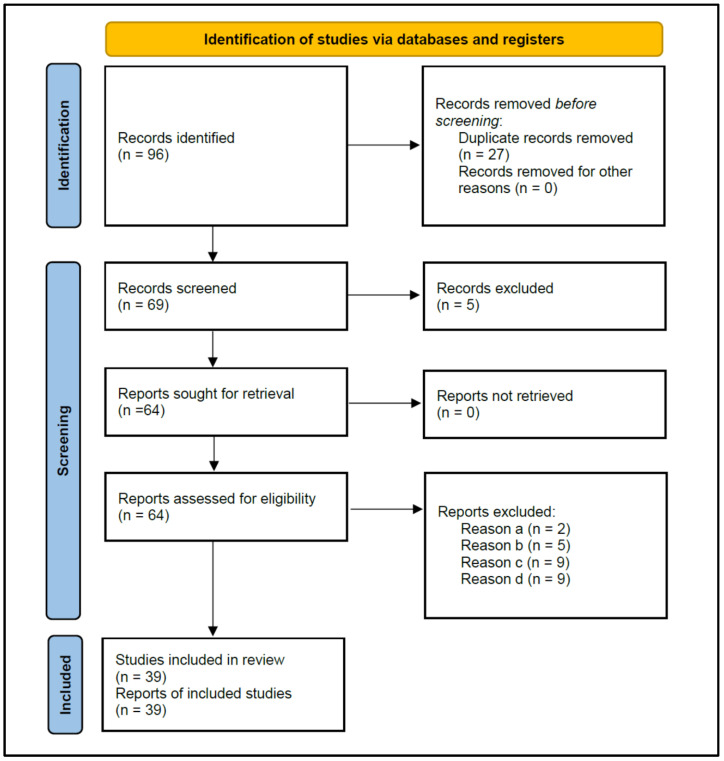
Flowchart of the search strategy, following the Preferred Reporting Items for Systematic Reviews and Meta-analyses (PRISMA) guidelines 2020.

Figure 1 shows the flowchart of the strategy followed to search for the studies selected for this review, according to PRISMA 2020 instructions. The initial search resulted in 96 published studies. After the removal of duplicate studies (n = 27), 69 studies were screened for titles and abstracts, and five that did not investigate antibiotic prescriptions were excluded, leaving 64 for reading the full text.

The eligibility criteria determined the exclusion of 25 studies (Table 1) for the following reasons: (a) Two studies did not allow data extraction on the antibiotic prescription habits of dentists [21,22]; (b) Five studies provided data from surveys carried out on dental students, rather than dentists [23,24,25,26,27]; (c) Nine studies did not provide data from surveys, but from medical records of patients treated with antibiotics [28,29,30,31,32,33,34,35,36]; and (d) Nine studies did not specify the diagnosis of the treated periapical pathology [37,38,39,40,41,42,43,44,45].

### 2.1. Characteristics of the Included Studies

Finally, 39 studies fulfilled the inclusion criteria and were selected for the systematic review and meta-analysis [13,15,16,17,18,19,20,46,47,48,49,50,51,52,53,54,55,56,57,58,59,60,61,62,63,64,65,66,67,68,69,70,71,72,73,74,75,76,77].

Table 2 shows the characteristics of the included studies (authors and year, country, number of respondents, and percentage of total and prescribers), together with the main outcomes, indicating the percentage of antibiotic prescriptions in each clinical situation. All studies were cross-sectional surveys and level 4 evidence, according to the Center for Evidence-Based Medicine at Oxford [78].

Regarding the prescribers, seven studies included in the sample only endodontists [18,50,52,55,59,73,74], two studies [20,76] included two separated samples, endodontists and general practitioners, considered as two different studies in the meta-analysis, one study included only dental surgeons [75], and the rest included general practitioners.

In most of the studies, the main outcomes were the percentage of antibiotic prescriptions in each of the four clinical situations of periapical endodontic disease. However, nine studies missed data regarding any of the clinical forms of apical periodontitis [46,58,62,63,65,66,67,68,70].

### 2.2. Meta-Analysis of Antibiotic Prescription in Symptomatic Apical Periodontitis

**Figure 2:**
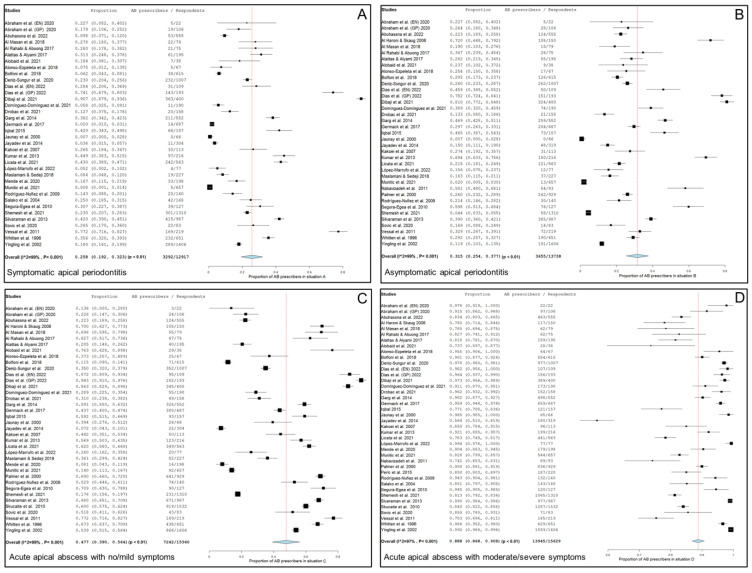
Forest plot of the included studies in the meta-analysis for prescription of antibiotics by general practitioners in the treatment of apical disease. (**A**) Symptomatic apical periodontitis; (**B**) asymptomatic apical periodontitis or chronic apical abscess; (**C**) acute apical abscess with no/mild symptoms; (**D**) acute apical abscess with moderate/severe symptoms.

Thirty-four studies, two of them considered doubly [20,76], including 12,917 respondents, provide data about the percentage of antibiotic prescriptions in cases of symptomatic apical periodontitis, no swelling and no/mild symptoms. Figure 2A shows the forest plot of the corresponding meta-analysis. The overall percentage of antibiotic prescriptions in this clinical situation was 25.8% (95% CI = 19.2–32.3%; *p* < 0.001). Heterogeneity value was I^2^ = 99.0% (*p* < 0.001).

### 2.3. Meta-Analysis of Antibiotic Prescriptions in Asymptomatic Apical Periodontitis or Chronic Apical Abscess

Thirty-five studies, two of them considered doubly [20,76], including 13,738 respondents, provide data about the percentage of antibiotic prescriptions in cases of a chronic apical abscess (asymptomatic apical periodontitis, sinus tract presence and no/mild symptoms). Figure 2B shows the forest plot of the corresponding meta-analysis. The overall percentage of antibiotic prescriptions in this clinical situation was 31.5% (95% CI = 25.4–37.7%; *p* < 0.001). Heterogeneity value was I^2^ = 99.0% (*p* < 0.001).

### 2.4. Meta-Analysis of Antibiotic Prescription in Acute Apical Abscess with No/Mild Symptoms

Thirty-seven studies, two of them considered doubly [20,76], including 15,360 respondents, provided data about the percentage of antibiotic prescriptions in cases of acute apical abscess with no/mild symptoms. Figure 2C shows the forest plot of the corresponding meta-analysis. The overall percentage of antibiotic prescriptions in this clinical situation was 47.7% (95% CI = 39.0–56.4%; *p* < 0.001). Heterogeneity value was I^2^ = 99.0% (*p* < 0.001).

### 2.5. Meta-Analysis of Antibiotic Prescription in Acute Apical Abscess with Moderate/Severe Symptoms

Thirty-eight studies, two of them considered doubly [20,76], including 15,629 respondents, provide data about the percentage of antibiotics prescriptions in cases of acute apical abscess with moderate/severe symptoms. Figure 2D shows the forest plot of the corresponding meta-analysis. The overall percentage of antibiotic prescriptions in this clinical situation was 88.8% (95% CI = 86.8–90.8%; *p* < 0.001). Heterogeneity value was I^2^ = 97.0% (*p* < 0.001).

### 2.6. Meta-Analysis of Antibiotics Prescription by Endodontists

**Figure 3:**
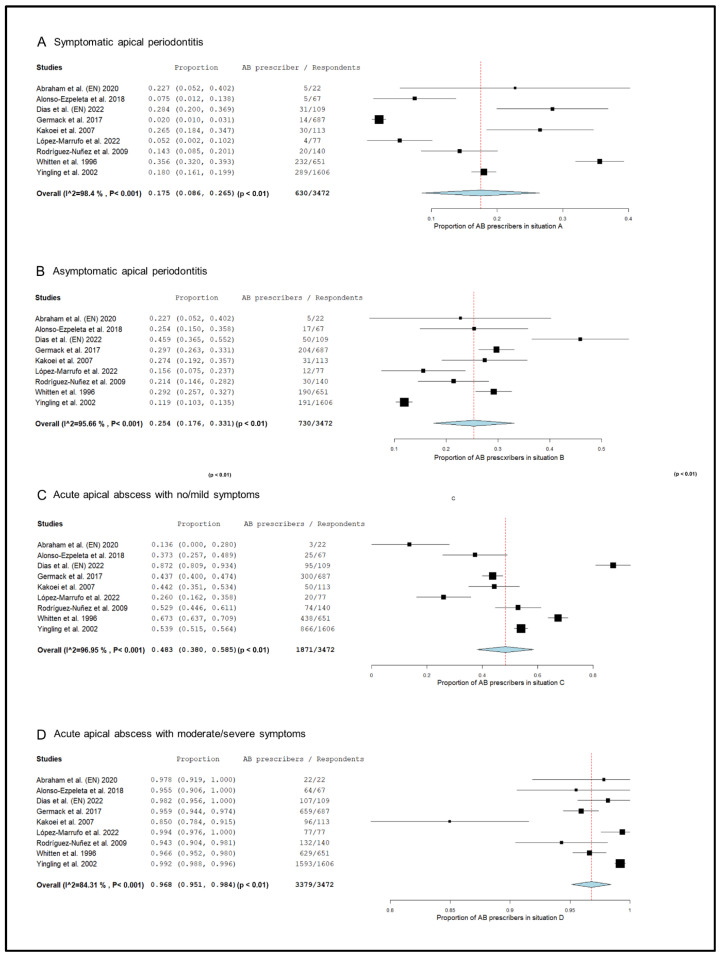
Forest plot of the included studies in the meta-analysis for prescription of antibiotics by endodontists in the treatment of apical disease. (**A**) Symptomatic apical periodontitis; (**B**) asymptomatic apical periodontitis or chronic apical abscess; (**C**) acute apical abscess with no/mild symptoms; (**D**) acute apical abscess with moderate/severe symptoms.

The prescription pattern of the subgroup of studies that included only endodontists [18,20,50,52,55,59,73,74,76], was analyzed (Figure 3). In symptomatic apical periodontitis, 17.5% (95% CI = 8.6–26.5%; *p* < 0.001) of endodontists prescribed antibiotics.

The percentage of endodontists who prescribed antibiotics in cases of asymptomatic apical periodontitis was 25.4% (95% CI = 17.6–33.1%; *p* < 0.001). In the case of acute apical abscess with no/mild symptoms, 48.3% (95% CI = 38.0–58.5%; *p* < 0.001) of endodontists prescribed antibiotics. Finally, 96.8% (95% CI = 95.1–98.4%; *p* < 0.001) of endodontists prescribed antibiotics in the treatment of acute apical abscesses with moderate/severe symptoms.

### 2.7. Risk of Bias

The risk of bias was evaluated for each study (Table 3). Twenty-three studies were classified as high risk of bias [13,16,17,18,19,20,46,49,50,52,53,54,56,58,59,61,66,67,68,69,71,74,76], thirteen had a moderate risk of bias [15,48,55,57,60,63,64,65,70,72,73,75,77], and three studies reported low risk of bias [47,51,62]. The sum of points assigned to the 39 included studies was 170 out of a possible maximum of 312. Therefore, the total risk of bias was considered moderate.

### 2.8. GRADE Assessment of the Certainty of Evidence

**Figure 4:**
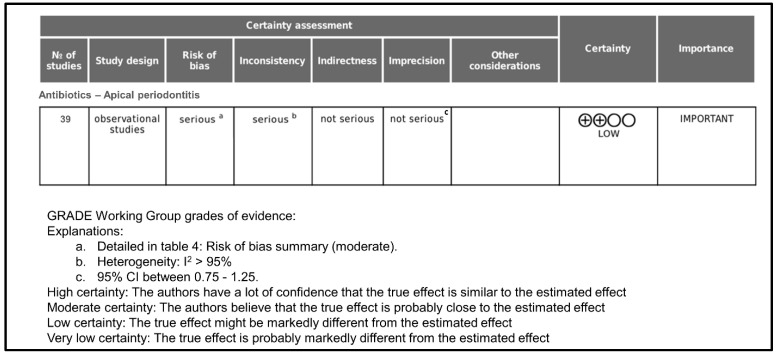
GRADE assessment of certainty level.

Figure 4 shows the GRADE assessment of certainty level. Initially, the certainty of evidence was rated as low since all included studies were cross-sectional surveys. Taking into account that the overall risk of bias was moderate, it was classified as serious.

Regarding inconsistency, it was rated as serious because, in all cases, heterogeneity was >95%. Indirectness was rated as not serious because all studies provide direct evidence about antibiotics prescription in apical periodontitis. Imprecision was rated as not serious because the 95% confidence intervals in all cases were between 0.75 and 1.25. As far as publication bias is concerned, it occurs when the publication of research results is influenced, in addition to the quality of the research, by the tested hypotheses and the importance and direction of the results. In the case of surveys on antibiotic prescription, in which the result is objective data, it has not been considered that publication bias could be important. There were no other considerations that modified the degree of certainty, and the final rating for the certainty of the evidence was low.

## 3. Discussion

The aim of this study was to analyze the global pattern of antibiotic prescription by dentists in the management of apical periodontitis. For this purpose, a systematic review and meta-analysis were performed, including the available evidence. The main conclusion was that, unfortunately, and generally, dentists inappropriately prescribe antibiotics in the treatment of apical periodontitis. More specifically, antibiotics are being overprescribed. In three of the clinical forms of apical periodontitis (in systemically healthy patients), it is not necessary to prescribe antibiotics; however, the percentages of dentists who prescribe them are important. Thus, in symptomatic apical periodontitis with no swelling and no/mild symptoms, in asymptomatic apical periodontitis with chronic apical abscess and sinus tract present, with no/mild symptoms, and in acute apical abscess with no/mild symptoms [6], the percentages of dentists prescribing antibiotics were 26%, 32%, and 48%, respectively. On the contrary, and surprisingly, in cases where antibiotics are indicated, such as acute apical abscess with swelling and moderate/severe symptoms [6,79], only 89% of dentists would prescribe antibiotics.

The present results show that endodontists prescribed antibiotics more properly than general practitioners. The specific postgraduate training on endodontic infections received by endodontists, as well as the greater experience in the management of apical periodontitis, may explain their more appropriate prescription of antibiotics. The percentages of endodontists prescribing antibiotics were considerably lower compared to general practitioners in cases of symptomatic apical periodontitis (18%) and in cases of asymptomatic apical periodontitis (25%). Also, in the treatment of acute apical abscess with moderate/severe symptoms, a case in which it is indicated to prescribe antibiotics, the percentage of endodontists prescribing antibiotics was 97%, almost 100%. On the contrary, the clinical form of apical periodontitis in which endodontists most inappropriately prescribed antibiotics was the acute apical abscess with no/mild symptoms. This is a clinical situation in which antibiotics are not indicated in systemically healthy patients [6,79], yet the percentage of endodontists who prescribed them (48%) was the same as that of general practitioners. What characterizes acute apical abscesses is swelling, and it seems that this is what alarms the clinicians and leads them to prescribe antibiotics, even in the absence of moderate/severe symptoms. But localized swelling with no/mild symptoms in non-medically compromised patients is a clinical situation in which antibiotics are not indicated [6]. Clinical evidence only supports antibiotics prescription in the management of apical periodontitis when the infection spreads systemically; the patient is febrile, or both [7,79]. When the patient has no swelling and moderate/severe symptoms, antibiotic use is not indicated. In these cases, the appropriate treatment should be limited to root canal treatment, with pulp extirpation, which eliminates the source of infection, followed by drainage of abscess, debridement of the root canal space and analgesics [5]. Given evidence of systemic involvement and manifest, rapid and diffuse spread of the infection, antibiotics should be prescribed [6]. However, the indication for antibiotics for the treatment of apical periodontitis changes radically when the patient is immunosuppressed or medically compromised. In these cases, antibiotics are indicated from the first phase of the acute apical abscess [6,79].

The search carried out for this systematic review provided a large number of studies, indicating the great interest of the scientific community in the topic. The design of all studies was cross-sectional surveys carried out among dentists, rated as having a low degree of certainty. Although the inconsistency was serious due to the high heterogeneity of the studies, the evaluation of a moderate risk of bias leads to not modifying the consideration of the quality of the overall evidence, using the GRADE methodology, as low certainty.

Although this study has included surveys carried out in countries around the world, the result may not be extrapolated to all countries. As can be concluded from Table 3, another astonishing conclusion of the present study is that there are no data about dentist’s antibiotic prescription patterns in the treatment of apical periodontitis in many countries, some of them with large populations, such as China, Russia, Indonesia, Germany, France, Argentine or Mexico. In these countries, it must be encouraged that adequate surveys are carried out to know the antibiotic prescription pattern of dentists in the treatment of endodontic infections.

In recent years, the challenge posed by the increase in bacterial strains resistant to antibiotics has led scientific societies, including ESE, AAE and ADA, to make recommendations on the indication of antibiotics in the treatment of endodontic infections [6,9,79]. Most of the studies included in the meta-analyses were conducted before these campaigns began, so perhaps prescribing antibiotics for the treatment of apical periodontitis is more appropriate today. In fact, recently published data demonstrate that these campaigns are working and that dentists tend to prescribe antibiotics more appropriately in the treatment of endodontic infections [52]. The antibiotic prescribing habits of Spanish endodontists have improved following the ESE’s awareness and positioning campaign on antibiotics in endodontics [13,52]. Even so, there is a percentage of dentists who still prescribe antibiotics incorrectly.

### Limitations and Strengths

Although undoubtedly one of the strengths of this review is the large number of studies included, with a total of responding dentists between 12,917 and 15,629, the presence of important limitations must also be noted. This systematic review includes cross-sectional studies carried out using surveys specifically designed to collect information relative to the patient’s conditions in which antibiotics were prescribed. Although the survey instrument has traditionally been effective in obtaining appropriate information on the practice of endodontics [39,80,81], these surveys generally have a response rate that is not very high, ranging from 30 to 45%. In the specific case of the studies included in this systematic review, the representativeness of the sample was low, with percentages of respondents below 50% in 11 of the 39 included studies. This has been taken into account in the risk of bias assessment, and so the final rating for the certainty of the evidence was low.

Another limitation of this study is that 14 of the studies are from more than 10 years ago, so the result obtained may not faithfully reflect the current situation of antibiotic prescription in apical disease.

## 4. Materials and Methods

### 4.1. Registry Protocol

To report this systematic review, the Preferred Reporting Items for Systematic Reviews and Meta-Analyses (PRISMA) guidelines [82] were used. A protocol was prospectively preregistered at the International Prospective Register of Systematic Reviews (PROSPERO) (CRD42023431788). The methodological guidance for systematic reviews of observational epidemiological studies reporting prevalence and cumulative incidence data [83] was followed to carry out this study.

### 4.2. Review Question

The research question was formulated following the CoCoPop (Condition, Context, and Population) mnemonic [83], as follows: What is the pattern of antibiotic prescription (Condition) by dentists (Population) in the treatment of the different clinical forms of apical periodontitis (Context).

The main outcome was the percentage of dentists prescribing antibiotics in the treatment of each clinical form of endodontic apical disease. The clinical forms of the apical disease were established according to previous studies [12,15,19,75] and the AAE classification [2], as follows: A: Symptomatic apical periodontitis, no swelling, no/mild symptoms; B: Chronic apical abscess/Asymptomatic apical periodontitis, sinus tract present, no/mild symptoms; C: Acute apical abscess, swelling, no/mild symptoms; and D: Acute apical abscess, swelling, moderate/severe symptoms.

### 4.3. Eligibility Criteria

All studies reporting the pattern of antibiotic prescription in the treatment of the different clinical forms of periapical disease were included. The inclusion criteria were as follows: (a) Cross-sectional surveys carried out among dentists; (b) Asking about antibiotic prescribing habits; (c) Inquiring about the treatment of the different clinical forms of periapical disease; (d) Studies specifying the diagnoses of the clinical forms of apical periodontitis.

The exclusion criteria applied are as follows: (a) Studies that did not report data about antibiotic prescription habits by dentists; (b) Studies that provided data from surveys carried out on dental students but not dentists; (c) Studies that did not provide data from surveys, but instead used medical records of patients treated with antibiotics; (d) Studies that did not specify the diagnosis of the treated periapical disease.

### 4.4. Search Strategy and Information Sources

Once the research question and the eligibility criteria were established, the search strategy was designed. A literature search was undertaken with no limits on time or language until May 2023 in PubMed-MEDLINE (1949–present), EMBASE (1949–present) and Scielo. The electronic search was carried out using the main descriptors cited in previous studies on this topic, combining Medical Subject Heading (MeSH) terms and text word (tw): (dental pulp diseases OR pulpitis OR dental pulp necrosis OR apical periodontitis OR periapical diseases OR periapical periodontitis OR periapical abscess) AND (antibiotic OR antibacterial agents) AND (dentist OR endodontist) AND (prescription OR inappropriate prescribing OR prescription drug misuse OR drug overuse OR prescription drug overuse) (Table S1; supplemental file). The references of all relevant articles, as well as those of selected studies, were hand-searched to locate cited studies that had not been found in the main search.

To select the studies, the titles and abstracts of the articles found were evaluated individually by three of the authors (J.J.S.-E., J.M.-G., and D.C.-B.). When the information obtained from the title and abstract was insufficient, the full text was also accessed. In the second phase, the full texts of all the selected articles were read, and the inclusion and exclusion criteria were applied. When there was disagreement regarding the inclusion of a study, it was resolved by consensus among the three authors. Logically, duplicate studies were only considered once. The information on the studies that matched the inclusion criteria was collected by these same three authors. A fourth author (J.J.S.-M.) solved disagreements. The information related to publication was extracted: article’s identification (authors and year of publication), number of respondents and percentage of responses, the country where the survey was conducted, and prescriber (general practitioner, endodontist and dental surgeon). The main outcome variables were the percentages of dentists who prescribed antibiotics in the treatment of each of the forms of periapical endodontic disease.

### 4.5. Risk of Bias Assessment

Each study was individually assessed for internal methodological risk of bias by three authors (M.L.-L., D.C.-B. and J.A.M.-M.). In case of discrepancy, the authors deliberated until they reached an agreement. The methodology used was based on the Newcastle–Ottawa Scale adapted for cross-sectional studies [84], with modifications of [85]. This scale was modified and adjusted for the outcome of interest. The items were grouped into two domains: sample selection (including representativeness of the sample, sample size and non-respondents) and assessment of the outcome. Scores were assigned as follows:

1. Sample selection (maximum of six points).
  1.1 Sample representativeness: It was evaluated based on the objective of the study. To study the prescription pattern of antibiotics in a particular country, the characteristics of the respondents to the survey should be in accordance with the population and chosen randomly.
    - Rightly representative of the average in the target population (all dentists or random sampling): three points.
    - Rather representative of the average in the target population (non-random sampling): two points.
    - Particular group: one point.
    - The sampling strategy is not described: no points.
  1.2 Sample size:
    - The sample size calculation is justified, and the size is satisfactory: one point.
    - Not justified size: no points.
  1.3 Non-respondents:
    - The response rate is >80%: two points.
    - The response rate is unsatisfactory: one point.
    - The response rate is not reported: no points.
2. Outcome (maximum of two points).
  2.1. Assessment of the outcome.
    - The proposed diagnoses used the periapical pathology nomenclature proposed by the AAE: two points.
    - The proposed diagnoses used a nomenclature similar to that of the periapical pathology proposed by the AAE: one point.
    - The proposed diagnoses used signs and symptoms without specifying the periapical pathology proposed by the AAE: no points.

The maximum score that a study can obtain is 8 points. A score of 0–4 points was considered a high risk of bias; a score of 5–6 points was considered a moderate risk of bias; and a score of 7–8 points was classified as a low risk of bias.

### 4.6. Data Extraction and Analysis

One of the authors (M.L.-L.) was responsible for data extraction, while three reviewers (J.A.M.-M., D.C.-B. and J.J.S.-E) confirmed the data to confirm the absence of errors and conducted the analysis of the articles; when there were discrepancies in any article, they were discussed until consensus was reached. To analyze and synthesize the data, the following information was extracted: author and year of publication, country, percentage of respondents, prescriber (general practitioner or endodontist), and percentage of antibiotic prescription in each clinical situation.

To determine the pattern of antibiotic prescription in each clinical form of periapical endodontic disease, a meta-analysis was performed with the OpenMeta Analyst version 10.10 software [86], using the DerSimonian–Laird method with the binary random effects model. Forest plots were produced to graphically represent the overall percentage of antibiotic prescriptions in each clinical form of apical periodontitis.

The Higgins I^2^ test was used to calculate the variance and heterogeneity among studies. Slight heterogeneity was considered if I^2^ was between 25 and 50%, moderate if the I^2^ value was between 50 and 75%, and high if >75% [87]. Finally, a *p*-value of 0.05 was considered significant.

### 4.7. Grading of Recommendations Assessment, Development and Evaluation

The Grading of Recommendations Assessment, Development and Evaluation (GRADE) tool was used to evaluate the certainty of evidence [88]. This tool calculates an initial level of certainty by assessing the design of the included studies. Then, different domains, such as the risk of bias, inconsistency, directionality, imprecision, publication bias, dose-response gradient, confounding factors, or effect size, are analyzed to determine a final level of certainty [89].

## 5. Conclusions

The worldwide pattern of antibiotic prescription by dentists in the treatment of endodontic infections is inappropriate and does not comply with the recommendations made by scientific societies and clinical guidelines. The vast majority of dentists adequately prescribe antibiotics for the treatment of acute apical abscess when it progresses and expands or when the patient shows systemic symptoms. However, a significant percentage of dentists also prescribe antibiotics to treat apical periodontitis when it is a localized problem and only requires drainage and root canal treatment. Given these results, it is advisable to maintain the campaigns initiated by scientific societies and propose new educational initiatives to promote the coherent and appropriate use of antibiotics in the management of apical disease.

## Figures and Tables

**Table 1:** Excluded studies and reasons for their exclusion.

Reason for Exclusion	Excluded Studies
Studies that did not allow extraction of data on the antibiotic prescription habits of dentists	Yu et al., 2020 [21] Kim et al., 2018 [22]
Studies that provided data from surveys carried out on dental students, rather than dentists	Jain et al., 2015 [23] Doshi et al., 2017 [24] Struzycka et al., 2019 [25] Salvadori et al., 2019 [26] Arican et al., 2021 [27]
Studies that did not provide data from surveys, but from medical records of patients treated with antibiotics	Kandemir et al., 2000 [28] Tanwir et al., 2015 [29] Asmar et al., 2016 [30] Lalloo et al., 2017 [31] Bjelovucic et al., 2019 [32] Al Asmar Ramli et al., 2020 [33] Alzahrani et al., 2020 [34] Carlsen et al., 2021 [35] Di Giuseppe et al., 2021 [36]
Studies that did not specify the diagnosis of the treated periapical pathology	Preus et al., 1992 [37] Mainjot et al., 2009 [38] Kaptan et al., 2013 [39] Bidar et al., 2015 [40] Buttar et al., 2017 [41] Madarati et al., 2018 [42] Roberts et al., 2019 [43] Khalil et al., 2022 [44] Vengidesh et al., 2023 [45]

**Table 2:** Included studies: authors and year, country, number of respondents and percentage of the total, and prescribers, together with the main outcomes and the percentage of antibiotic prescriptions in each clinical situation.

Authors and Year	Country	Respondents (% of Total)	P	Antibiotic Prescription in Each Clinical Situation (%)			
				A	B	C	D
Whitten et al., 1996 [74]	USA	651 (43.4%)	EN	35.6	29.2	67.3	96.6
Jaunay et al., 2000 [57]	Australia	66 (61%)	GP	0	0	39.4	98.5
Palmer et al., 2000 [66]	UK	929 (60.1%)	GP	-----	26	69.6	90
Yingling et al., 2002 [73]	USA	1606 (50.1%)	EN	18	11.9	53.9	99.2
Salako et al., 2004 [68]	Kuwait	168 (84%)	GP	25.0	------	-----	85.1
Al Haroni & Skaug 2006 [46]	Yemen	150 (53.6%)	GP	-----	72	70	78
Kakoei et al., 2007 [59]	Iran	113	EN	26.5	27.4	44.2	85.0
Rodríguez-Núñez et al., 2009 [18]	Spain	140 (31.1%)	EN	14.3	21.4	52.9	94.3
Segura-Egea et al., 2010 [75]	Spain	127 (64%)	DS	30.7	59.8	70.9	94.5
Skucaite et al., 2010 [70]	Lithuania	1532 (53.8%)	GP	-----	------	60	84
Nabavizadeh et al., 2011 [65]	Iran	93 (77.5%)	GP	-----	581.721	-----	74.2
Vessal et al., 2011 [72]	Iran	219 (48.6%)	GP	-----	32.9	77.2	75.3
Kumar et al., 2013 [60]	India	216 (87.8%)	GP	44.9	69.4	56.9	92.1
Sivaraman et al., 2013 [69]	USA	987 (21%)	GP	42	39	68	99
Garg et al., 2014 [54]	India	552 (34.5%)	GP	38.2	46.9	59.1	90.2
Jayadev et al., 2014 [58]	India	304 (79.8%)	GP	3.6	15.0	7.2	56.4
Iqbal 2015 [56]	Saudi Arabia	157 (78.5%)	GP	42.0	46.5	59.2	77.1
Peric et al., 2015 [67]	Croatia	220 (50%)	GP	-----	------	-----	85.0
Alattas & Alyami 2017 [48]	Saudi Arabia	195 (98%)	GP	31.3	28.2	20.5	81.5
AlRahabi & Abuong 2017 [47]	Saudi Arabia	75 (85%)	GP	28.0	34.7	62.7	82.7
Germack et al., 2017 [55]	USA	687 (22.9%)	EN	6.4	29.7	43.7	95.9
Al Masan et al., 2018 [16]	UK	79 (60%)	GP	27.8	19.0	71.9	78.5
Alonso-Ezpeleta et al., 2018 [50]	Spain	67 (91.2%)	EN	7.5	25.4	37.3	95.5
Bolfoni et al., 2018 [15]	Brazil	615 (4.4%)	GP	6.2	20.5	11.5	90.1
Maslamani & Sedeji 2018 [62]	Kuwait	227 (75.6%)	GP	8.4	16.3	36.1	-----
Abraham et al., 2020 [76]	UAE	106	GP	17.9	26.4	22.6	91.5
Abraham et al., 2020 [76]	UAE	22	EN	22.7	22.7	13.6	100
Deniz-Sungur et al., 2020 [17]	Turkey	1007 (5.6%)	GP	23.0	26	35	97
Mende et al., 2020 [63]	Lithuania	198 (93.0%)	GP	16.7	------	8.1	90.4
Sovic et al., 2020 [71]	Croatia	83 (92%)	GP	26.5	16.9	51.8	85.5
Alobaid et al., 2021 [49]	Saudi Arabia	38 (63.3%)	GP	18.4	23.7	76.3	73.7
Dibaji et al., 2021 [51]	Iran	400 (100%)	GP	90.7	81.0	86.2	97.3
Domínguez-Domínguez et al., 2021 [13]	Spain	190 (95%)	GP	5.8	39.0	28.9	91.1
Drobac et al., 2021 [53]	Serbia	158 (25.2%)	GP	12.7	13.3	31.0	96.2
Licata et al., 2021 [61]	Italy	563 (52.6%)	GP	43	21.5	62	78.3
Šimundić-Munitic et al., 2021 [64]	Croatia	657 (24.0%)	GP	0.8	2.0	14.0	82.8
Abuhassna et al., 2022 [77]	Saudi Arabia	555 (61.1%)	GP	9.5	22.3	52.4	83.4
Dias et al., 2022 [20]	Colombia	193 (60.3%)	GP	74.1	78.2	94.3	96.4
Dias et al., 2022 [20]	Colombia	109 (34.1%)	EN	28.4	45.9	87.2	98.2
López-Marrufo et al., 2022 [52]	Spain	77 (77%)	EN	5.2	15.6	26.0	100
Shemesh et al., 2022 [19]	Israel	1310 (17.5%)	GP	23.0	4.4	17.6	81.3

P: Prescriber; GP: general practitioner. EN: endodontist. DS: dental surgeon. A: Symptomatic apical periodontitis, no swelling, no/mild symptoms. B: Asymptomatic apical periodontitis, sinus tract, no/mild symptoms (chronic apical abscess). C: Acute apical abscess, swelling, no/mild symptoms. D: Acute apical abscess, swelling, moderate/severe symptoms.

**Table 3:** Quality assessment of individual studies and risk of bias. The maximum total score with the sum of the 39 studies would be 312. Each * is one point.

	Selection			Outcome	
Authors and Year	Representativeness of the Sample (Maximum: 3)	Sample Size Calculation (Maximum: 1)	Non-Respondents (Maximum: 2)	Assessment (Maximum: 2)	Risk of Bias
Whitten et al., 1996 [74]	*		*	**	High
Jaunay et al., 2000 [57]	***		*	*	Moderate
Palmer et al., 2000 [66]			*	*	High
Yingling et al., 2002 [73]	*	*	*	**	Moderate
Salako et al., 2004 [68]	*		**	*	High
Al-Haroni & Skaug 2006 [46]	**		*		High
Kakoei et al., 2006 [59]	*			*	High
Rodríguez-Núñez et al., 2009 [18]	*		*	**	High
Segura-Egea et al., 2010 [75]	***		*	**	Moderate
Skucaite et al., 2010 [70]	**	*	*	*	Moderate
Nabavizadeh et al., 2011	***	*	*	*	Moderate
Vessal et al., 2011 [72]	**	*	*	*	Moderate
Kumar et al., 2013 [60]	*		**	**	Moderate
Sivaraman et al., 2013 [69]	*		*	**	High
Garg et al., 2014 [54]	**		*	*	High
Jayadev et al., 2014 [58]			**	**	High
Iqbal et al., 2015 [56]	*		*	*	High
Peric et al., 2015 [67]	*			*	High
Alattas & Alyami 2017 [48]	**		**	**	Moderate
AlRahabi & Abuong 2017 [47]	***		**	**	Low
Germack et al., 2017 [55]	**		*	**	Moderate
Al Masan et al., 2018 [16]			*	**	High
Alonso-Ezpeleta et al., 2018 [50]	*			**	High
Bolfoni et al., 2018 [15]	**	*	*	**	Moderate
Maslamani & Sedeqi 2018 [62]	***	*	*	**	Low
Abraham et al., 2020 [76]	*		*	**	High
Deniz-Sungur et al., 2020 [17]	**	*	*		High
Mende et al., 2020 [63]	**		*	**	Moderate
Sovic et al., 2020 [71]	*		**	*	High
Alobaid et al., 2021 [49]			*	*	High
Dibaji et al., 2021 [51]	***		**	**	Low
Domínguez-Domínguez et al., 2021 [13]			**	**	High
Drobac et al., 2021 [53]	*		*	*	High
Licata et al., 2021 [61]	*		*	*	High
Šimundić-Munitic et al., 2021 [64]	**	*	*	**	Moderate
Abuhassna et al., 2022 [77]	**	*	*	**	Moderate
Dias et al., 2022 [20]	*	*	*	*	High
López-Marrufo et al., 2022 [52]	*		*	**	High
Shemesh et al., 2022 [19]	*		*	**	High
TOTAL	57	10	44	59	170 (Moderate)

## Data Availability

Not applicable.

## Supplementary Materials

The following supporting information can be downloaded at https://www.mdpi.com/article/10.3390/antibiotics13040289/s1, Table S1. Lists MeSH and keyword combinations used for the search strategy.
