# Supplementary material for: Antibiotic Over-Prescription by Dentists in the Treatment of Apical Periodontitis: A Systematic Review and Meta-Analysis
Source: Antibiotics (Basel). 2024 Mar 22;13(4):289. doi: 10.3390/antibiotics13040289 (PMC11047734; doi:10.3390/antibiotics13040289)
Supplement: Supplementary file 1 [file antibiotics-13-00289-s001.zip › antibiotics-2913883-supplementary.pdf]

antibiotics

MDPI

Supplementary file

Antibiotic Over-Prescription by Dentists in the Treatment of Apical Periodontitis: A Systematic Review and Meta-Analysis

Juan A. Méndez-Millán, María León-López, Jenifer Martín-González, Juan J. Saúco-Márquez, Daniel Cabanillas-Balsera * and Juan J. Segura-Egea *

Department of Stomatology (Endodontic Section), School of Dentistry, University of Sevilla, C/Avicena s/n, 41009 Sevilla, Spain; jamendezmillan@hotmail.com (J.A.M.-M.); maria.leon.lopez.98@gmail.com (M.L.-L.); jmartin30@us.es (J.M.-G.); jjsauco@us.es (J.J.S.-M.)

* Correspondence: dcabanillas@us.es (D.C.-B.); segurajj@us.es (J.J.S.-E.)

Table S1. Lists MeSH and key words combinations used for the search strategy.

(("anti-bacterial agents"[Pharmacological Action] OR "anti-bacterial agents"[MeSH Terms] OR ("anti-bacterial"[All Fields] AND "agents"[All Fields]) OR "anti-bacterial agents"[All Fields] OR "antibiotic"[All Fields]) OR ("anti-bacterial agents"[Pharmacological Action] OR "anti-bacterial agents"[MeSH Terms] OR ("anti-bacterial"[All Fields] AND "agents"[All Fields]) OR "anti-bacterial agents"[All Fields] OR ("anti"[All Fields] AND "bacterial"[All Fields] AND "agents"[All Fields]) OR "anti bacterial agents"[All Fields])) AND (("dentists"[MeSH Terms] OR "dentists"[All Fields] OR "dentist"[All Fields]) OR ("dentists"[MeSH Terms] OR "dentists"[All Fields] OR "endodontist"[All Fields])) AND (("prescriptions"[MeSH Terms] OR "prescriptions"[All Fields] OR "prescription"[All Fields]) OR ("inappropriate prescribing"[MeSH Terms] OR ("inappropriate"[All Fields] AND "prescribing"[All Fields]) OR "inappropriate prescribing"[All Fields]) OR ("prescription drug misuse"[MeSH Terms] OR ("prescription"[All Fields] AND "drug"[All Fields] AND "misuse"[All Fields]) OR "prescription drug misuse"[All Fields]) OR (drug[All Fields] AND overuse[All Fields]) OR ("prescription drug overuse"[MeSH Terms] OR ("prescription"[All Fields] AND "drug"[All Fields] AND "overuse"[All Fields]) OR "prescription drug overuse"[All Fields])) AND (("dental pulp diseases"[MeSH Terms] OR ("dental"[All Fields] AND "pulp"[All Fields] AND "diseases"[All Fields]) OR "dental pulp diseases"[All Fields]) OR ("pulpitis"[MeSH Terms] OR "pulpitis"[All Fields]) OR ("dental pulp necrosis"[MeSH Terms] OR ("dental"[All Fields] AND "pulp"[All Fields] AND "necrosis"[All Fields]) OR "dental pulp necrosis"[All Fields]) OR ("periapical diseases"[MeSH Terms] OR ("periapical"[All Fields] AND "diseases"[All Fields]) OR "periapical diseases"[All Fields]) OR ("periapical periodontitis"[MeSH Terms] OR ("periapical"[All Fields] AND "periodontitis"[All Fields]) OR "periapical periodontitis"[All Fields]) OR ("periapical abscess"[MeSH Terms] OR ("periapical"[All Fields] AND "abscess"[All Fields]) OR "periapical abscess"[All Fields]) OR ("periapical periodontitis"[MeSH Terms] OR ("periapical"[All Fields] AND "periodontitis"[All Fields]) OR "periapical periodontitis"[All Fields] OR ("apical"[All Fields] AND "periodontitis"[All Fields]) OR "apical periodontitis"[All Fields]))
